# Self-reported height and weight: timeliness and source of the participant information

**DOI:** 10.1186/s13690-025-01742-w

**Published:** 2025-10-15

**Authors:** Anja Schienkiewitz, Almut Richter, Gert B.M. Mensink, Julika Loss

**Affiliations:** Department of Epidemiology and Health Monitoring, Robert Koch Institute, Nordufer 20, 13353 Berlin, Germany

**Keywords:** Self-report, Body height, Body weight, Adults, National health interview survey, GEDA 2022, Germany

## Abstract

**Background:**

Self-reported data on body height and weight have often been used as an alternative when standardized measurements in large population-based examination surveys were not feasible. The timeliness and source of self-reported body height and weight information is often unknown. The aim of this analysis was to understand how up-to-date the self-reported data on body height and weight are, and from which source this information is given.

**Methods:**

Within the population based national health interview survey “German Health Update” (GEDA 2022) data were collected from 06 to 10/2022 from 1729 women and 1436 men aged 18 years and older (mean age: 58.8 years, SD: 17.6 years). Participants were asked when height and weight had last been measured, how this was obtained and if they possess a scale. For the response categories given for these questions, the proportions (%) with 95% confidence intervals (CI) were presented. A weighting factor was used to correct for different selection probabilities and for deviations of the sample from the German population. Differences were considered statistically significant if the calculated *p*-value is < 0.05.

**Results:**

Among adults in Germany, 45.9% (95% CI: 43.1–48.7%) of the population reported that their height had been measured in the last year, 35.0% (32.3–37.8%) more than a year ago and 19.1% (17.1–21.3%) more than 10 years ago. 54.8% (52.0–57.6%) stated that they had been measured by a doctor/medical staff, 19.8% (17.7–22.1%) reported the value on their identity card and 20.3% (18.1–22.6%) had measured themselves. 67.2% (64.5–69.7%) of the population responded that they had measured their weight in the last 4 weeks and 11.9% (10.2–13.8%) in the last 3 months. 84.0% (81.6–86.2%) had a scale at home, and of these 85.9% (83.5–88.1%) had also measured their weight with this scale.

**Conclusion:**

Self-reported data on height measurements, on average are much less recent than weight measurements, which appear to be relatively up-to-date. This limitation should be considered when BMI analyses are based on self-reported data. More precise information on height and weight can only be determined with standardized up-to-date measurements.

**Table:** 

**Text box 1. Contributions to the literature**
• Self-reported height and weight data are frequently used when standardized measurements are not available e.g. due to high data collection costs.
• It is well studied that self-reports are often biased due to overestimation of height and underestimation of weight, but it is unknown to what extent self-reports on body height and weight are up-to-date and where this information comes from.
• Self-reported data on height measurements are on average much less recent than weight measurements, which appear to be relatively up-to-date.
• When discussing and interpreting the results of monitoring overweight and obesity, potential inaccuracies must be taken into account.

## Background

To obtain accurate data for monitoring and surveillance of overweight and obesity, a direct measurement of body height and weight is preferred. However, self-reported data on body height and weight have often been used as an alternative, as standardized measurements in examination surveys are time-consuming and cause higher data collection costs. This predominantly involves costs for study logistics (e.g. equipment to conduct the body measurement) and organizing research staff, who carry out the standardized measurements, as well as the necessary quality control of the study process and collected data. Furthermore, conducting studies in research centers are also time-consuming for participants which can reduce the willingness to participate. Unfortunately, self-reported data can lead to misreporting, with individuals typically overestimating body height and underestimating body weight, resulting in underestimated Body Mass Index (BMI), lower average BMI reported for populations, and an underestimated prevalence of overweight and obesity [1, 2]. The degree of misreporting often varies among individuals according to age, sex, and weight status: The extent of underestimation of BMI varies between men and women with a range of no difference to around 2 kg/m^2^, and large differences between studies [1]. According to age groups, it is evident that older adults tend to overestimate their height but body weight is generally more accurate [1]. Reporting accuracy is also influenced by current weight status: men and women with overweight or obesity tend to underreport their weight, whereas people with underweight tend to overreport their weight [1, 2]. The extent to which the biases in self-assessment of height and weight have changed over time is unclear: there are indications that both bias of self-reported height and weight has declined between 1988 and 2008 [3] or remained stable among men and women over time [4]. Research findings demonstrate that individuals from low education groups were more prone to provide inaccurate information on their body weight [5] and the agreement between self-reported and objectively measured values of height and weight was lower among individuals with less education [6].

In the literature, there are different reasons that may underlie misreporting of weight and height, such as social norms [7, 8], social desirability [9], and lack of knowledge [10] since height measurements are seldomly conducted and, if they are, it may be unknown how a self-assessment of height should be preferably carried out. Social norms mean that self-assessment of weight and size is influenced by people’s ideas about their ideal body image. Social desirability leads to giving a weight and/or height value that corresponds to a social norm for weight and/or height, and endeavoring to make a good impression, even if the given value itself is inaccurate. Lack of knowledge about one’s own body measures is another alternative explanation for misreporting of weight and height.

Although influencing parameters regarding validity and reliability of body height and weight have been investigated, studies or data on timeliness and sources of self-reported height and weight are lacking. Mean annual weight gain among adults in Germany aged 45–64 years was about 250 g for men and 240 g for women [11]. This could add up to several kilos over a decade. Furthermore, seasonal and holiday weight gain is well documented in international literature [12]. Therefore, it is important to collect data that is as up-to-date as possible. In Germany, it is assumed that adult height loss begins at about the age group 50–59 years [13] but other studies also observed changes from the age of 30 onwards [14] and therefore, lacking recent height measurements are a concern. To assess BMI and prevalence of overweight and obesity using data from interview, paper/pencil or online surveys, it is important to be able to judge if the BMI can be reliably based on participants’ reporting. Therefore, this paper presents aspects that help understand the validity of self-reported information on body height and weight from participants of a population-based telephone survey: last time and source of information of height and weight measurement as well as the possibility (e.g. equipment such as scales) to measure body weight at home. The literature is lacking data on these aspects, and until now the proportion of people who could measure their body weight on private scales is unknown. The option to use self-measurements should also be considered, although the (valid) standardized measurement of body height is more complicated from a scientific point of view than measuring weight, as this can hardly be done alone, and is usually not often carried out among adults. Therefore, the source of height information is also relevant.

The aim of the study was to determine the source of self-reported body height and weight and how up-to-date this information is. From a public health perspective, it is important to obtain valid information for the population as a whole, including certain populations groups, so the study will examine whether there are differences between gender, age and education groups.

## Methods

### Study design

The ‘German Health Update’ (GEDA, ‘Gesundheit in Deutschland aktuell’) is a cross-sectional population-based survey of the residential population in Germany and is part of the national monitoring system conducted by the Robert Koch Institute [15]. GEDA aims to collect data through telephone interviews on health status, access to health care, health-related behavior, and demographic as well as socioeconomic determinants. GEDA 2022 uses a programmed, structured questionnaire (Computer Assisted Telephone Interview, CATI) and was conducted from January till December 2022. The sample is selected using a dual-frame approach, contacting respondents via both landlines and mobile phones. The random selection of participants from households with more than one person was done according to the so-called Swedish key [16] to ensure representativeness of the German adult population aged 18 years and older. Data on timeliness and source of participant information on self-reported body height and weight were collected between June and October 2022. The present analysis includes a representative sample of the population (*n* = 3178) aged 18 years and older living in private households in Germany.

GEDA 2022 is subject to strict compliance with the data protection regulations of the EU General Data Protection Regulation (GDPR) and the German Federal Data Protection Act (BDSG). The Ethics Committee of Charité-Universitätsmedizin Berlin has assessed the ethical aspects of the study and approved the implementation (application number EA2/201/21). Study participation was voluntary. All participants were informed about the aim and content of GEDA 2022 as well as the data protection. Their informed consent to participate in the interview survey was obtained verbally.

### Anthropometric variables – key variables

In GEDA 2022, the question on body height was: “*How tall are you when you don’t wear shoes?*” The information was given in centimeter (cm). To find out when the last measurement took place the participants were asked “*And when did you have your last measurement or when were you last measured?*” The three answer options were: “Last year”, “More than a year ago”, “More than 10 years ago”. To get information about body height, the question “*What is the source of the height information*?” was asked with the following answer options “I have been measured by a physician/medical staff”, “Height information is from my identity card”, “I have measured myself”, “other”. The question on body weight was “*How much do you weigh when you don’t wear clothes and shoes? Please indicate your body weight in kilogram (kg). Pregnant women*,* please indicate your weight before pregnancy*.” To find out when body weight was measured the last time, the question was “*When did you weigh yourself or when were you last weighed?*”. The answer options were “in the last 4 weeks”, “in the last 3 months”, “in the last six months”, “in the last year”, “more than a year ago”, or “more than 10 years ago”. Since very few persons (< 1%) chose the last category “more than 10 years ago” we combined the last two categories to “more than a year ago”. Participants were also asked if they have a scale that they use to measure body weight (“*yes/no*”) and if they measured their above-mentioned body weight with this scale (“*yes/no*”).

### Covariates

The survey questionnaire covered sex registered at birth (options: female/male) and gender identity (options: female/male/other). To describe gender differences, gender identity was used [17]. Based on information on the highest achieved education level, educational status was defined using the Comparative Analysis of Social Mobility in Industrial Nations (CASMIN) scale which includes information on general and vocational training and was classified into the education groups: low, medium, and high [18].

From individual body height and weight BMI was calculated as the ratio of weight to the square of height (kg/m^2^). World Health Organization (WHO) cut-off criteria were applied to define BMI groups for normal weight (BMI < 25.0 kg/m^2^), overweight (BMI 25.0–<30.0 kg/m^2^), and obesity (BMI ≥ 30.0 kg/m^2^) [19].

### Statistical analysis

For descriptive analyses, proportions were reported with 95% confidence intervals (95% CI) by gender (women, men), age group (18–29 years, 30–44 years, 45–64 years, ≥ 65 years) and education group (low, medium, high). The different numbers of missing data (people don‘t know the answer, are not sure or give no response) for the variables last measurement of height (*n* = 71), source of height information (*n* = 94), last measurement of weight (*n* = 10), scale owner (*n* = 1), measurement with scale (*n* = 23) resulted in different number of cases for each outcome.

Multinomial logistic regression models were used to analyze the independent effect of gender (reference: men), age group (reference: 30–44 years), education (reference: medium education group) and BMI group (reference: normal weight BMI < 25.0 kg/m²) on the dependent variable (1) the time of last height measurement (*n* = 3078; categories ‘last year’ (reference group), ‘more than one year ago’, and ‘more than 10 years ago’) (2), the source of height information (*n* = 3038; categories ‘doctor/medical staff’, ‘identity card’ (reference group), ‘self-measurement’, ‘other’) and (3) the time of last weight measurement (*n* = 3104; categories ‘last 4 weeks’ (reference group), ‘last 3 months’, ‘last six months’, ‘last year’, ‘more than one year ago’). Multivariable logistic regression models were used to analyze the ownership of a scale (yes/no) independent of age, gender and education. Odds ratios (OR) were calculated as effect estimates with 95% confidence intervals (95%-CI).

In order to correct for deviations in the sample from the population structure in Germany, all analyses were performed using a weighting factor. The weighting factor was applied to correct for different selection probabilities, included (1) a design weighting to adjust for the various selection probabilities (mobile service and landline) and (2) an adaptation to the official population figures based on age, sex, federal state, district type (as of: 31.12.2020 [20]), and education [21]. The sampling weight improved representativeness of results from this sample for the total population. Further details can be found here [15].

All statistical analyses were performed using survey procedures for complex samples in SAS 9.4 (SAS Institute, Cary, NC). Group differences were tested for significance using the Rao-Scott chi-square test for complex samples corrected by the F distribution. A statistically significant difference between the groups is assumed when the probability of error (p), which is calculated in consideration of the weighting and of the survey design, is less than 0.05.

## Results

Table 1 presents the characteristics of the study participants: from 3178 participants aged 18 years and older (1729 women, 1436 men, mean age: 58.8 years, SD: 17.6 years), thirteen individuals had a different gender identity or did not provide information, 20 provided no information on education, 3090 participants had valid data for body weight, 3172 for body height, and 3088 for BMI.

**Table 1:** Study characteristics

	Number of cases	Unweighted proportion	Weighted proportion	Missing values
	*n*	%	%	*n*
**Total**	3178	100	100	
**Gender**				
Women	1729	54.6	51.0	13*
Men	1436	45.4	49.0	
**Age group**				
18–29 years	238	7.5	16.0	0
30–44 years	486	15.3	22.4	
45–64 years	1194	37.6	34.6	
≥ 65 years	1260	39.7	27.0	
**Education group**				
Low	526	16.7	27.2	20
Medium	1349	42.7	52.9	
High	1283	40.6	19.9	
**BMI group**				
< 25.0 kg/m²	1458	47.2	46.6	90
25.0–<30.0 kg/m²	1113	36.0	34.9	
≥ 30.0 kg/m²	517	16.7	18.5	

* different gender identity or no information: participants were not considered in the sex stratified analysis

Table 2 shows the prevalence of *time of last body height measurement*: 45.9% (95%-CI: 43.1–48.7%) of the respondents stated that their height was measured in the last year, 35.0% (32.3–37.8) stated that the measurement was taken more than one year ago, and 19.1% (17.1–21.3) stated that the measurement was taken more than 10 years ago. There were no statistically significant differences between women and men and education groups. A measurement of body height in the last year reported nearly 50% of the youngest and oldest age group, whereas the proportion among the 30–44 and 45–64 years old was slightly lower (40.5% and 44.6%, respectively). A measurement more than a year ago was mentioned more often among older age groups (45–64 years, ≥ 65 years) compared to the younger age groups (18–29 years, 30–44 years). More than 10 years ago was reported more often by the age groups of 30 years and older compared to the youngest age group of 18–29 years.

**Table 2:** Proportion of time of last height measurement (%, 95%-CI) according to gender, age and education group

	Last year	More than one year ago	More than 10 years ago	*p*-value
	% (95%-CI)	% (95%-CI)	% (95%-CI)	
**Total**	45.9 (43.1–48.7)	35.0 (32.3–37.8)	19.1 (17.1–21.3)	
**Gender**				*0.10*
Women	43.9 (40.2–47.7)	34.7 (31.1–38.4)	21.4 (18.6–24.6)	
Men	48.4 (44.3–52.6)	34.7 (30.7–38.9)	16.9 (14.2–20.0)	
**Age group**				*< 0.0001*
18–29 years	48.1 (39.6–56.7)	44.0 (35.6–52.6)	8.0 (4.5–13.8)	
30–44 years	40.5 (34.2–47.2)	41.7 (35.3–48.4)	17.7 (13.9–22.4)	
45–64 years	44.6 (40.2–49.1)	30.4 (26.4–34.8)	25.0 (21.2–29.1)	
≥ 65 years	50.5 (46.2–54.9)	30.0 (26.1–34.3)	19.4 (16.1–23.2)	
**Education group**				*0.14*
Low	45.3 (39.4–51.4)	31.9 (26.5–37.8)	22.8 (18.1–28.2)	
Medium	47.0 (43.0–51.0)	36.2 (32.3–40.4)	16.8 (14.2–19.7)	
High	43.6 (39.7–47.7)	35.7 (31.9–39.7)	20.6 (17.7–23.9)	

The multinomial analysis indicated that, independent of education group and BMI group, women (OR = 1.44; 95%-CI: 1.06–1.96) and age groups 30–44 years (2.52; 1.21–5.24) and 45–64 years (2.96; 1.47–5.95) had higher odds ratios for height measurement more than 10 years ago compared to 18-29-year olds. The effect can also be observed in the age group ≥ 65 years, although the OR was not statistically significant (1.93; 0.95–3.89).

Table 3 presents the prevalence of *source of height measurement*. A measurement by a doctor/medical staff was reported by 54.8% (52.0–57.6%), an indication from the identity card by 19.8% (17.7–22.1%), a self-measurement by 20.3% (18.1–22.6%), and other sources by 5.1% (3.9–6.7). There were no statistically significant differences between women and men, but between age groups and education groups. A measurement of body height by a doctor or medical staff was stated more often in the age groups 45–64 years and ≥ 65 years than in both younger age groups, whereas a self-report from the identity card was given more often in the youngest age group than in the oldest age group. A self-measured body height was reported by a higher proportion of the younger age groups compared to older age groups. Whereas nearly two third of men and women in the low education group (63.0%) and only 46.8% in the high education group reported a body height measurement by a doctor or medical staff, the proportion of participants who retrieved their body height from the identity card did not differ statistically significant between education groups. A self-measurement is mentioned only by 14.5% in the low education group compared to one quarter in the high education group.

The multinomial analysis confirmed that, independent of gender, education group and BMI group, older age groups such as those aged 45–64 years (OR = 1.94; 95%-CI: 1.15–3.27) and 65 years and older (2.97; 1.76–5.01) had higher odds ratios for measurement by a doctor or medical staff, education was not significantly associated with the source of height information.

**Table 3:** Proportion of source of height measurement (%, 95%-CI) according to gender, age and education group

	Measurement			Other	*p*-value
	by doctor or medical staff	from the identity card	by self-measurement		
	% (95%-CI)	% (95%-CI)	% (95%-CI)		
**Total**	54.8 (52.0-57.6)	19.8 (17.7–22.1)	20.3 (18.1–22.6)	5.1 (3.9–6.7)	
**Gender**					*0.51*
Women	53.7 (49.9–57.5)	21.3 (18.3–24.6)	19.5 (16.7–22.6)	5.5 (3.8–7.9)	
Men	55.6 (51.4–59.8)	18.2 (15.2–21.6)	21.4 (18.1–25.1)	4.8 (3.2–7.1)	
**Age group**					*p* < 0.0001
18–29 years	42.2 (33.9–51.0)	27.3 (20.5–25.4)	27.5 (20.6–35.7)	3.0 (1.2–7.3)	
30–44 years	41.6 (35.3–48.2)	21.5 (16.7–27.2)	29.5 (23.8–36.0)	7.3 (4.3–12.1)	
45–64 years	59.5 (55.0-63.7)	19.9 (16.5–23.7)	15.5 (12.9–18.5)	5.2 (3.4–7.8)	
≥ 65 years	67.5 (63.3–71.4)	13.8 (11.3–16.8)	14.3 (11.7–17.3)	4.5 (2.6–7.4)	
**Education group**					*p* < 0.0001
Low	63.0 (57.0-68.6)	16.2 (12.5–20.7)	14.5 (10.6–19.4)	6.3 (3.8–10.5)	
Medium	53.7 (49.6–57.8)	20.9 (17.7–24.6)	21.2 (18.1–24.8)	4.1 (2.7–6.1)	
High	46.8 (42.9–50.8)	21.9 (18.8–25.5)	25.1 (21.7–28.9)	6.1 (3.8–9.6)	

Regarding *body weight*, 67.2% (64.5–69.7%) of the population answered having measured their weight in the last 4 weeks, 11.9% (10.2–13.8%) in the last 3 months, 8.6% (7.1–10.4%) in the last 6 months, 4.3% (3.4–5.5%) in the last year, and 8.1% (6.6–9.8%) more than a year ago. There are no statistically significant differences between women and men, between age groups or education groups related to time of last measurement. Considering the possibility to measure the body weight at home, 84.0% (81.6–86.2) reported that they have a scale. With 86.4% (83.4–88.8), women reported more often to have a scale that they use to measure body weight at home compared to men (81.4% (77.5–84.9), *p* = 0.03). Older age groups more often had a scale compared to younger age groups (18–29 years: 76.7% (68.7–83.2); 30–44 years: 81.2% (75.5–85.8); 45–64 years: 85.0% (80.8–88.4); ≥65 years: 89.5% (85.9–92.2); *p* = 0.003). No statistically significant differences between education groups were observed. Of all adults who owned a scale to measure body weight, 85.9% (83.5–88.1) reported that they have measured the mentioned body weight with this scale. There were neither statistically significant differences between women and men nor between age groups, but men and women from the high education group (91.3% (89.0-93.2)) reported more often a measurement of the body weight with their own scale compared to participants from the low education group (82.8% (76.9–87.5); *p* = 0.02).

## Discussion

### Principal findings

When asked about their height in this survey, about half of the respondents referred to a recent measurement which has taken place within the last year. Around one fifth (19.1%) of respondents gave a height that has been measured more than 10 years ago, this percentage was highest in the age group 45–65 years. Self-measurement of height played a minor role: most respondents reported a height that has been determined by medical staff. The rate of measurement by health professionals rises with increasing age, probably reflecting that height measurements in medical offices were being taken more frequently as patients get older and height begins to decline in that age [13, 14]. Still, about a quarter to a fifth of the participants under 65 years simply gave the value that is documented in their identity card. It could be expected that body weight was measured more often than that they measure their height: in fact, two thirds of respondents referred to a weight that has been measured within the last 4 weeks. Only 8% of respondents gave a value even though they have not been weighed in a year or longer. The vast majority of participants owned a scale at home, the percentage getting higher with increasing age; mostly, the weight they gave has been measured with this scale.

### Comparison with other studies

A reliability study with data from USA, Australia, UK and 14 European countries found that people who were most likely to give inconsistent and unreliable answers about their body height were older and had lower levels of education [10]. Although we could not validate the height information, a possible reason could be a lack of up-to-date information in these population groups. In our study, nearly 20% of people aged 65 years and older were more likely to provide height data more than a decade ago and younger people did not tend to refer to more recent height data. It seems obvious that individuals aged 18–29 years were less likely to have a measurement older than 10 years, as they would have still been growing during their last decade of life. However, these age group differences cannot be observed for the body weight measurement. Furthermore, the timeliness of the information on body height did not differ among education group, although Evans et al. shows that men and women with lower levels of education reported significantly different height values when asked twice [10]. The reasons for this relationship are unclear. Therefore, findings regarding education groups needs to be further investigated.

In addition, older respondents were more likely to have their height measured by a health professional, which could be advantageous on the one hand for the precision of height value and on the other hand for an up-to-date height value as height changes with age. A reason can be that older persons more often visit their physician and are more likely to attend voluntary health checks in primary care in Germany [22], and therefore are also more likely to cite the height information from these check-ups. In the age group ≥ 65 years 20% reported that the last height measurement was more than 10 years ago. 50% reported a measurement in the last year. It still remains unclear if older people who visited the physician had regularly height measurements. Probably up-to-date anthropometry is only performed if there is a reason (e.g. illness/disease). It is described in the literature that women who annually visit a physician were more accurate in their reporting of height and weight [23]. As measurements were not available, unfortunately, it was not possible to make any further statements about the accuracy of the self-declarations made in our data. In Germany, information on height is part of the information on the identity card. However, when applying for an identity card, height is often based on self-reports. Only in certain cases, there is a measurement taken on site, e.g. if the information appears implausible or the person does not know their height. Since it is usually transferred from the previous identity card it may be outdated. Therefore, the quality is probably lower than from a recent self-measured value.

It is plausible that people did not measure their body height as often as they weigh themselves, given the higher changeability of weight and the difficulties of precise self-measurement of height. In our survey, 25% of respondents 45–64 years and almost 20% of respondents 65 years and older referred to a height measurement more than 10 years ago. This seems rather problematic as body height decreases with age. A literature review of 16 studies with data from participants up to 80 years of age showed that men lost an average in height of 5 cm and women 6 cm from the age of 30 years [14]. Therefore, there is a risk that height data is imprecise and often overreported in a substantial subgroup of survey participants when only based on self-reports [24]. This could lead to imprecise BMI calculations and an underestimation of overweight and obesity prevalence in higher age groups. Epidemiological studies may therefore benefit from a better public awareness of body height trajectories over the life course, and from a better access to professional height measurements. In Germany, men and women aged 35 years and older with statutory health insurance were eligible for medical height and weight measurement every three years, as part of the voluntary “health checks” [25]. Precise knowledge of one’s own height can therefore be improved by increasing the participation rates of health check-ups, e.g. through better information and motivation.

So far, the given value for body weight was more up-to-date compared to body height without any differences between women and men, between age groups or education groups. Data from the Third National Health and Examination Survey (NHANES III, 1988–1994) indicated that women 60 years and older reported a body weight closer to the measured value compared to younger women [24]. Four out of five men and women answered to have a scale at home, and of those, four out of five reported the weight information measured with this scale. Unfortunately, it remains unclear how the remaining 20%, who did not have scales, derived their body weight. Although the majority of respondents had their own scales at home, a social gradient is obvious: men and women from the high education group reported more often a measurement with an own scale compared to participants from the low education group. There is evidence that positive health behaviors are more common among persons with high education in Germany [26]. Regarding body weight, a survey conducted by the Health Knowledge Foundation indicated that people with higher socioeconomic status (SES) attach more importance to weight control, compared to respondents with a low SES [27]. This social gradient must be considered when collecting information on body weight from different populations. Social desirability has often been considered in response behavior, but it is apparent that some parts of the population have no actual knowledge of their body height and weight and therefore cannot answer precisely.

### Strengths and limitations

To our knowledge, this is the first study that analyzed the time of last measurement of height and weight and the possibility of weight measurement at home. Although measurement of body height and weight is preferable and the gold standard to assess objective values, self-report is a cost-effective and practical approach for several reasons, and will probably play an important role in the future, e.g. in online surveys. Beyond that, to ask about body height and weight will be a regular component of the German Health Panel, so it is necessary to know more about the quality of this data [28].

However, we should be aware of some limitations. A limitation of the study is that we cannot ruled out a potential selection bias within specific groups: the complex weighting procedure improved the representativity for the given characteristics and was used to make population-based conclusions. Another limitation is that there were only very general answer categories for last measurement of body weight without further differentiation. These were therefore not suitable for estimating possible changes, especially as these can differ significantly from person to person. Another limitation is that the self-reports on body height and weight data could not be further verified using objective measurement methods. To correct for bias in self-reported height and weight, prediction equations could be applied, but this requires measurements from a subpopulation. For better comparison with the measured values, data on body height and weight from a previous German health survey (2003) were adjusted using a correction formula [17]. A similar procedure could be used in future assessments. Nevertheless, in the absence of standardized measurements and beyond misreporting of weight and height due to social norms, social desirability and lack of knowledge, self-reports are useful. Half of the respondents reported a height measurement in the last year and two thirds a weight measurement in the last four weeks. In conclusion, weight information for this adult population is probably fairly up-to-date.

### Recommendation

When collecting self-reported height and weight data, it would be desirable that this information was measured by a healthcare professional shortly beforehand. If this is not possible, it might be useful to have instructions and guidance for participants on how to measure height and body weight at home, e.g. using an own scale. Most importantly seems to be that the self-report is based on a recent measurement before the survey. For instance, women reported weight and height reasonably reliable and accurate in the Sister Study, where participants were informed about a weight and height measurement during a home visit and may have reported an up-to-date weight and height in the computer-assisted telephone interview and the self-administered questionnaires [29]. Because GEDA 2022 was conducted as a telephone interview, it was not possible to provide instruction information on measuring oneself in advance, but for future surveys, especially if they are conducted online, information could be obtained from measurements taken at home. Whether participants would really measure their body weight directly before the online assessment has to be investigated. From our data we can conclude that the majority of men and women in the population would be able to measure their weight because they have a scale. Self-measurement of height is considered challenging as professional technical equipment is not usually available at home and other measurement methods are subject to possible inaccuracies. Participants should be informed in the announcement of the study that they will be asked to provide their height. Therefore, measurements taken by medical professionals that are as recent as possible should be sought. Alternatively, the suitability of self-measurement at home should be tested in further studies. Furthermore, in the discussion and interpretation of self-reported data on height and weight, researchers should consider possible limitations and varying degrees of these limitations across different population groups.

## Conclusion

In the absence of objective measures, which are of course the gold standard to assess anthropometric parameters and therefore preferable, self-reported weight and height from surveys are frequently used because they are time and cost efficient. While self-reported data on body height and weight provide a practical approach to monitor overweight and obesity, it comes with inherent challenges due to potential inaccuracies. Whereas among adults in Germany the last height measurement was taken considerably longer ago, the information on weight appears to be relatively up-to-date.

These limitations must be considered when analyses of BMI are based on self-reported data. More precise information on height and weight can only be determined with standardized up-to-date measurements.

## Data Availability

The authors state some access restrictions applying to the data. The dataset cannot be made publicly available, because the informed consent of the study participants did not cover the public deposition of the data. However, the minimum dataset underlying the results is archived at the Research Data Center at the Robert Koch Institute and can be accessed by researchers on reasonable request. On-site access to the data set is possible at the Secure Data Center of the Robert Koch Institute. Requests can be sent by e-mail to fdz@rki.de.
